# Development and Evaluation of Microencapsulated Oregano Essential Oil as an Alternative Treatment for *Candida albicans* Infections

**DOI:** 10.1021/acsami.4c07413

**Published:** 2024-07-27

**Authors:** Liliana Fernandes, Ainara Barco-Tejada, Elena Blázquez, Daniela Araújo, Artur Ribeiro, Sónia Silva, Lorena Cussó, Sofia Costa-de-Oliveira, M. Elisa Rodrigues, Mariana Henriques

**Affiliations:** † Centre of Biological Engineering, University of Minho, Campus de Gualtar, 4710-057 Braga, Portugal; ‡ Departamento de Bioingeniería, Universidad Carlos III de Madrid, 126, 28903 Getafe, Madrid, Spain; § Unidad de Medicina y Cirugía Experimenta, Instituto de Investigación Sanitaria Gregorio Marañón, 28029 Madrid, Spain; ∥ National Institute for Agrarian and Veterinary Research, Vairão, 4485-655 Vila do Conde, Portugal; ⊥ LABBELS − Associate Laboratory, 4710-057 Braga, Portugal; # Advanced Imaging Unit, Centro Nacional de Investigaciones Cardiovasculares Carlos III (CNIC), 28029 Madrid, Spain; g CIBER de Salud Mental, Instituto de Salud Carlos III, 28029 Madrid, Spain; h Division of Microbiology, Department of Pathology, and Center for Health Technology and Services Research − CINTESIS@RISE, Faculty of Medicine, University of Porto, 4200-450 Porto, Portugal

**Keywords:** Vulvovaginal candidiasis, *Candida albicans*, Vaginal microflora, Phytotherapeutic applications, Keratin microcapsules, Biofilm treatment

## Abstract

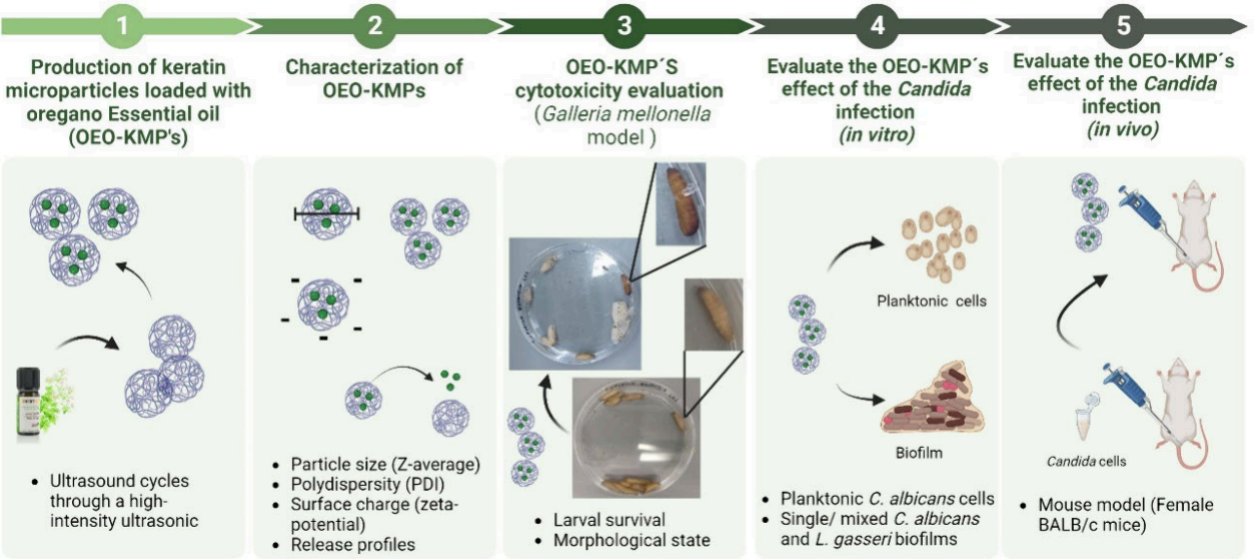

Vulvovaginal candidiasis (VVC) is characterized as a very common fungal infection that significantly affects women’s health worldwide. Essential oils (EOs) are currently being evaluated as an alternative therapy. The development of efficient techniques such as micro- or nanoencapsulation for protecting and controlling release is essential to overcome the limitations of EO applications. Therefore, the aim of this study was to develop and characterize oregano EO-loaded keratin microparticles (OEO-KMPs) as a potential treatment for VVC. OEO-KMPs were produced using high-intensity ultrasonic cycles and characterized in terms of morphological and physicochemical parameters. *In vitro* evaluation included assessing the toxicity of the OEO-KMPs and their effect against *Candida albicans* using microdilution and agar diffusion, while the activity against biofilm was quantified using colony forming units (CFU). The efficacy of the OEO-KMPs in an *in vivo* VVC mouse model was also studied. Female BALB/c mice were intravaginally infected with *C. albicans*, 24 h postinfection animals were treated intravaginally with 15 μL of OEO-KMPs and 24 h later vaginal fluid was analyzed for *C. albicans* and *Lactobacillus* growth (CFU mL^–1^). The results showed the stability of the OEO-KMPs over time, with high encapsulation efficiency and controlled release. This nanoparticle size facilitated penetration and completely inhibited the planktonic growth of *C. albicans*. In addition, an *in vitro* application of 2.5% of the OEO-KMPs eradicated mature *C. albicans* biofilms while preserving *Lactobacillus* species. In *in vivo*, a single intravaginal application of OEO-KMPs induced a reduction in *C. albicans* growth, while maintaining *Lactobacillus* species. In conclusion, this therapeutic approach with OEO-KMPs is promising as a potential alternative or complementary therapy for VVC while preserving vaginal microflora.

## 1 Introduction

Vulvovaginal candidiasis (VVC) is a common fungal infection that affects women and can be manifested at different stages of life, with peaks during pregnancy or when the immune system is weakened.^1^ At the beginning of VVC infection, patients may experience itching, redness, and burning of the vulva and vaginal mucosa, leading to frequent urination, dyspareunia, dysuria, and a consequent impaired quality of life.^1^*Candida albicans* is the most commonly found species, accounting for approximately 20% to 90% of the recovered isolates.^2^ One of the main virulence factors of *C. albicans* is the yeast-to-hypha transition, which occurs after adhesion to the epithelial cells of the vulvovaginal mucosa. Subsequent invasion by hyphae activates epithelial signaling pathways leading to pro-inflammatory responses and biofilm formation.^1^ The eradication of fungal biofilms requires critical medical intervention, including antifungal treatment through different routes of drug administration.^3^ Presently, azole antifungals are the main choice for preventing and treating invasive fungal infections. However, these agents have several limitations and disadvantages, including drug resistance and adverse effects especially in its long-term use that is associated with hepatotoxicity and hormone-related effects, including gynecomastia, alopecia, oligospermia, azoospermia, decreased libido, impotence and hyponatremia.^4^ In this context, natural products are currently being evaluated for their antimicrobial activity in order to develop alternative therapies, with lower costs, fewer adverse reactions and less negative impact.^5^ Previous studies have reported that essential oils (EOs) and their vapor phase (VP-EOs) exhibit antifungal activities against *Candida* species, having biocompatibility and low cost.^6−8^ Oregano EO has been shown to have anticandidal properties.^6,9^ Fernandes et al.^6^ demonstrated high antifungal activity of VP of OEO (VP-OEO), with a significant reduction in *C. albicans* and *Candida glabrata* biofilms, affecting membrane integrity and metabolic activity. However, EOs’ activity depends on several factors such as photosensitivity, high volatility, low water-miscibility, and degradability at high temperature, which reduces their bioavailability. Therefore, micro- or nanoencapsulation has been investigated as an effective technique to protect, control and prolong the release of EOs, improve the water-solubility and bioavailability of lipophilic compounds, and minimize the inconvenience caused by possible EOs’ side effects.^10,11^ Drug delivery systems, such as liposomes or nanoliposomes, nanoemulsions, films, or nanogels, protein-based nanoparticles have unique functionalities and offer promising applications in biomedical and materials sciences. These nanoparticles stand out for several reasons: their permeation and retention effect enable easy absorption and retention in target tissues; the amphiphilicity of proteins facilitates interaction with both the drug (hydrophilic or hydrophobic) and the solvent, thereby enhancing drug release efficiency; and the ease of surface modification allows for controlled drug binding and release.^12,13^ Additionally, protein nanoparticles provide greater *in vivo* stability during storage and postadministration. They are also relatively easy to prepare and control in terms of particle size, which is crucial for ensuring efficient and targeted drug delivery.^12,13^ In this sense, several types of protein can be used for fabricating microcapsules, such as keratin.^14^ Keratin is a natural protein found in different sources such as wool, human hair, nails, feathers and horns or hooves.^15,16^ This compound is a promising option for building drug carriers due to its biodegradability, biocompatibility, absorbability, nonimmunogenicity and reduction sensitivity.^12,17^ Moreover, due to a cysteine-rich structure and the presence of several functional groups, such as amid, carboxyl or sulfhydryl, it can be easily modified with biomolecules to improve its solubility, stability and can be applied in a wide range of biomedical and biotechnological applications.^15^ In this context, keratin-based drug carriers have been explored, including micro- or nanoparticles, nanogels or films.^16^

The aim of this work was to produce and characterize keratin microparticles (KMPs) loaded with oregano essential oil (OEO-KMPs) and to evaluate the anti-*Candida* activity of the OEO-KMPs using *in vitro* and *in vivo* assays. The effect of the OEO-KMPs on *Lactobacillus* species, beneficial bacteria in the vaginal microflora, was also evaluated. Within this context, the OEO encapsulation in microcapsules is anticipated to present a promising alternative to conventional treatments for VVC. This approach provides protection, controlled and prolonged release, maximizing the potential effect of the OEO.

## 2 Materials and Methods

### 2.1 Oregano Essential Oil Encapsulated into Microparticles of Keratin (OEO-KMPs)

#### 2.1.1 Oregano Essential Oil and Conditions

In this study, oregano EO (*Origanum compactum*, florame, France) (100% pure) was encapsulated in keratin nanoparticles. The OEO was stored in a desiccator at room temperature and was protected from light.

In this work, two synthetic fluids were used: simulated vaginal fluid (SVF) and simulated sweat fluid (SSF). The SVF was prepared according to Fernandes et al.^9^ and SSF was adapted from the international standard ISO 105-E01:2013^18^ and consisted of 6.25 g L^–1^ of NaCl (Biochem Chemopharma, France), 0.625 g L^–1^ of l-histidine monohydrochloride monohydrate (Sigma-Aldrich) and 2.5 g L^–1^ disodium hydrogen orthophosphate anhydrous (Merck- Sigma-Aldrich), the pH was adjusted to 5.5 with acetic acid (Fisher chemical).

#### 2.1.2 Keratin Extraction and Purification

Keratin was extracted from natural wool samples, gently provided by Dra Isabel Gouveia from the Department of Textile Science and Technology, Beira Interior University (Portugal). The wool samples were washed according to the IAEA/RL/50 1978 instructions to remove contaminants and lipids. The washing process involved several steps: first, the samples were washed with distilled water and acetone, with agitation at 250 rpm for 20 min, in four alternating cycles; then, the wool samples were subjected to a bleaching process using a kit, followed by thorough washing until all residues were removed; and after this, the samples were kept at 37 °C overnight.

The protocol, adapted from Tinoco et al.,^14^ was followed for keratin extraction, and it consisted of a solution that was prepared using urea (8 M), sodium metabisulfite (0.5 M), and sodium dodecyl sulfate (SDS) (0.2 M). The volume-to-mass ratio of dry hair (in grams) was consistently held at 10:1. Afterward, the mixture underwent heating at 100 °C for 30 min. Subsequent steps included incubation at 37 °C with agitation at 500 rpm overnight, followed by centrifugation at 4000 rpm for 15 min, and the resulting supernatant was subsequently filtered. The keratin solution obtained was dialyzed for 5 days using a dialysis membrane with a 14 kDa cutoff (Merk-Sigma), with the distilled water being changed twice daily. After 5 days, the solution was centrifuged for 15 min at 4000 rpm, and, as in the previous step, the resulting supernatant was filtered. The quantification of protein concentration was performed using the DC method (Bio-Rad), according to the manufacturer’s instructions.^19^ In this process, a yield of 30% was obtained. Subsequently, keratin was lyophilized for subsequent use.

#### 2.1.3 Preparation of the Keratin Microparticles for Encapsulation of Oregano Essential Oil

Keratin protein solutions were prepared with phosphate buffer saline (PBS 1×) pH 7.4 to a final concentration of 10 mg mL^–1^ with 5% (v/v) of OEO. For the homogenization process, the samples were subjected to ultrasound cycles (total time: 6 min; cycles: 8 and 2 s; amplitude: 40%) by a high-intensity ultrasonic equipment (SONICS, Vibra-Cell). The free OEO was separated from the particle’s formulations, by size-exclusion chromatography using a 5 kDa of cutoff PD-10 Desalting Column (GE-Healthcare), according to manufacturer’s protocol.^20^

#### 2.1.4 Determination of OEO-KMPs’ Encapsulation Efficiency

The encapsulation efficiency (EE) of the OEO within the keratin microparticles was measured using a standard calibration curve of the OEO in ethyl acetate. This curve was obtained using the maximal peak absorbance values of different OEO concentrations, measured by a UV–vis spectrophotometer (275 nm) (Synergy H1; BIOTEK). Then the free OEO was extracted from the aqueous phase with ethyl acetate and analyzed by spectrophotometry. Each sample was assayed in triplicate. The EE of OEO was evaluated by the difference between the initial oil concentration in the sample and the free oil concentration after encapsulation (eq 1).^14^
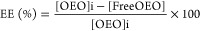
1

#### 2.1.5 Physicochemical Properties of the OEO-KMPs

The physicochemical properties (mean size diameter, polydispersity index (PDI) and surface charge (ξ-potential)) of the OEO-KMPs were measured using a Zetasizer Nano ZS (Malvern Instruments) at 25 °C. The samples were measured with a 1:10 dilution with PBS (1×) and read in triplicate. The solutions of the OEO-KMPs were kept protected from light and stored at 4 °C and their stability was monitored for 5 months. The results of this assay are presented as the mean ± standard deviation.^14^

#### 2.1.6 Evaluate the *in Vitro* Release of OEO

To measure the amount of OEO released, standard calibration curves in SVF and SSF were obtained by using the maximal peak absorbance values of different OEO concentrations prepared with SVF and SSF, using a UV–vis spectrophotometer. The *in vitro* release of OEOs for 72 h was accomplished using a dialysis membrane (14 kDa cutoff). For this, 2 mL of the solution of OEO-KMPs’ was placed inside the dialysis membrane and incubated in 15 mL of SVF or SSF, with agitation (250 rpm) at room temperature and in dark conditions. At specific time intervals, 200 μL of the SVF/SSF solution was withdrawn in triplicate and spectrophotometrically analyzed by an absorption reading at 250 nm. The amount released (%) was determined by comparing the released oil with the initial concentration of OEO encapsulated (eq 2).
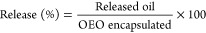
2

The stability of OEO-KMPs in interaction with SVF or SSF were also evaluated, encompassing an analysis of their physicochemical properties, including average diameter, PDI and surface potential ξ, as previously described.^14^

#### 2.1.7 OEO-KMPs’ Toxicity

To assess the *in vivo* skin toxicity of the OEO-KMPs, the *Galleria mellonella* model was used. The *G. mellonella* larvae were maintained on a diet based on pollen grains at 25 °C in the dark and carefully selected at the final stage of development when the weight was approximately 250 mg, as described previously by Araújo et al.^21^ Then, 5 μL of OEO-KMPs (17.88 mg mL^–1^), was spread over the *G. mellonella* larvae body (*n* = 10) and two control groups were carried out under the same conditions, one with PBS (*n* = 10) and the other with the empty keratin solution (*n* = 10). The three sets of larvae were kept at 37 °C and in dark conditions. The *G. mellonella* health index (movement, cocoon formation and melanization) and larvae survival were monitored over 72 h. The results were presented as the percentage of survival (%) and the health index. The experiment was performed in two independent assays.

### 2.2 Evaluate the OEO-KMPs’ Effect on the *Candida* Infection *in Vitro* Assays

#### 2.2.1 Microorganisms and Culture Conditions

This study used *Candida albicans* SC 5314 obtained from the American Type Culture Collection (ATCC) and *Lactobacillus gasseri* ATCC 33323 (acquired from DSMZ). The *C. albicans* was subcultured from a frozen stock (Sabouraud dextrose broth (SDB; Liofilchem) medium with 20% (v/v) glycerol (Biochem Chemopharma), at −80 ± 2 °C onto Sabouraud dextrose agar (SDA; Liofilchem) plates and incubated for 24 h at 37 °C.

The *L. gasseri* was kept in Mann Rogosa and Sharpe broth (MRSB; Liofilchem) with 20% (v/v) glycerol at −80 ± 2 °C. This species was subcultured from the frozen stock onto MRSB (1% of cells) and incubated for 72 h at 37 °C and 5% CO_2_ environment in saturated humidity.

#### 2.2.2 OEO-KMPs’ Effect on Planktonic *C. albicans* Cells

The inhibitory effect of OEO-KMPs on the growth of *C. albicans* was evaluated by the agar disk diffusion method, as described previously by Tran et al.^22^ Briefly, the SDA surface was inoculated using a swab dipped in a cell suspension adjusted to 1 × 10^8^ cells mL^–1^. After the inoculum was dried, a sterile filter paper disk (6 mm) (Liofilchem) was impregnated with 25 μL of the OEO-KMPs. Plates with disks containing only keratin and without the OEO-KMPs were also included as controls. Incubation of all plates occurred at 37 °C, and the diameters (mm) of the zones surrounding the disks were measured within 24 h.

The broth microdilution method according to CLSI M27-A4 guidelines was followed, and some modifications were introduced to adapt the method to the specific needs of this study. These modifications include adjustments in cellular concentrations and the specific mixing ratio of solutions, aiming to optimize the interaction between the compound and fungal cells and obtain more relevant results for evaluating the effectiveness of the OEO-KMPs. Briefly, different cell densities of *C. albicans* were tested, a lower (1 × 10^5^ CFU mL^–1^) and a higher (1 × 10^8^ CFU mL^–1^) concentration, in SVF and dispensed into 96-well round-bottom microtiter plates with the OEO-KMPs’ solution, both in a proportion of 1 (OEO-KMPs): 2 (cell concentration). Positive controls (*C. albicans* suspension) and negative controls (SVF or keratin solution) were included. Microtiter plates were incubated at 37 °C for a duration of 24 h. It was not possible to compare the turbidity of the solution between the conditions, as described in CLSI M27-A4, since OEO-KMPs is an opaque liquid. Then, each well was subcultured onto SDA plates and incubated for 24 h at 37 °C and the number of grown colonies (CFUs) was counted. The results were expressed as log CFU mL^–1^.

Both experiments were performed in triplicate with three independent assays.

#### 2.2.3 OEO-KMPs’ Effect on Single and Mixed *C. albicans* and *L. gasseri* Biofilms *in Vitro*

The effect of the OEO-KMPs on single and mixed *C. albicans* and *L. gasseri* biofilms was evaluated. Biofilms were developed as described by Stepanović et al.^23^ Briefly, the preinoculum of *C. albicans* was prepared by transferring a few colonies into SDB and incubating the culture for 18 h at 37 °C under agitation 120 rev/min agitation (120 rev/min) and *L. gasseri* was precultured in MRSB with 1% (v/v) of cells taken from the subculture during 24 h at 37 °C in an environment of 5% CO_2_ in saturated humidity. Then, both cellular suspensions were centrifuged and washed twice with PBS (5000 g for 10 min at 4 °C).

To study the effect in single *C. albicans* and *L. gasseri* biofilms, 1 mL of standardized suspension (1 × 10^7^ cells mL^–1^ prepared in SVF) of *C. albicans* and *L. gasseri* strains, separately, was transferred to a 24-well plate. For mixed-species cultures, 500 μL of *C. albicans* suspension was combined with 500 μL of *L. gasseri* suspension, and both suspensions were adjusted for 2 × 10^7^ CFU mL^–1^. The 24-well plates were incubated (24 h at 37 °C, 120 rev/min). Then, several concentrations of OEO-KMPs (0.09, 0.23, 0.45, 0.89, 2.682, 4.47 mg mL^–1^) were applied, for an additional 24 h. As positive control, biofilms were formed without any contact with the OEO-KMPs while SVF was used as negative control. The number of cultivable cells in the single and dual biofilms was calculated using the CFU counting methodology. For this, the biofilms were washed with PBS to remove planktonic fraction, and the suspensions with biofilm cells were serially diluted in PBS and then passed to SDA for single biofilm, SDA supplemented with 20 mg L^–1^ of gentamycin and MRSA supplemented with 1 mg L^–1^ of amphotericin B for dual biofilms. The plates were incubated (5% CO_2_ environment, at 37 °C for 24 h), and the number of colonies grown was counted and translated into CFU per milliliter (Log (CFU mL^–1^)).

### 2.3 Evaluation of the Effect of the OEO-KMPs in a Mouse Model

#### 2.3.1 Microorganism Culture

*Candida albicans* SKCA23-ACTgLuc, strain well characterized and widely used in animal models of VVC, was grown on SDA chloramphenicol plates (Condalab, Spain) overnight at 37 °C prior to the experiments. The inoculum with a concentration of 1.3 × 10^8^ CFU mL^–1^ in PBS was made by selecting two to three colonies from the 24 h culture of *C. albicans* SKCA23-ACTgLuc in SDA and adjusting with the Neubauer chamber.

#### 2.3.2 *Candida albicans* Infection and the Treatment with OEO-KMPs

Twenty-one BALB/c mice (Charles River) of 6–8 weeks and 19.29 ± 2 g were housed under a 12-h light–dark cycle at 23 °C ± 1 °C and 50% ± 5% humidity in the animal facility of Hospital General Universitario Gregorio Marañón, Madrid, Spain (ES280790000087). All animal procedures were conformed to EU Directive 2010/63 EU and national regulations (RD 53/2013) and were approved by the HGUGM. Animal Experimentation Ethics Committee, the local Ethics Committees, and the Animal Protection Board of the Comunidad Autónoma de Madrid (PROEX 083/18). All animals were allowed access to food and water and libitum.

Mice were divided into two groups: infected untreated (*n* = 10) and infected treated with OEO-KMPs (*n* = 11). Briefly, mice were injected with 17β-Estradiol (E2) 0.5 mg mL^–1^ dissolved in sesame oil (Sigma-Aldrich, USA), commonly used to dissolve the lipophilic substance E2. After 72 h, 2 × 10^6^ cells of *C. albicans* in 15 μL of PBS were inoculated into the vagina, as described by Relloso et al.^24^ Following a 24 h infection period, 15 μL of OEO-KMPs containing an OEO concentration of 17.88 mg mL^–1^ (for the treated group) or PBS (for the untreated group) were administered intravaginally. Microbiological cultures were then obtained from vaginal lavage and vaginal tissue samples 24 h after the application of the OEO-KMPs or PBS (used as a control). Vaginal lavage was performed by gently irrigating the vaginal vault with 50 μL of sterile PBS. This process was repeated four times, utilizing a 100 μL pipet tip, as described in previous studies.^24,25^ Following the lavage, serial dilutions (1:10, 1:100, and 1:1000) from the vaginal fluids were performed. These dilutions and the undiluted sample were subsequently incubated onto two types of agar plates: SDA and MRSA, for *C. albicans* and *Lactobacillus* species quantification, respectively. The agar plates were subsequently incubated for 24 h at 37 °C. After incubation, the CFUs were counted to determine the microbial content in the samples. In all the procedures, animals were awake except at the final point, when mice were anesthetized with 3% sevoflurane in 100% oxygen for vaginal lavages and sacrificed for the removal of vaginas for histological studies. Over time, the body weight of each animal was also monitored, an important parameter that allows the detection of side effects of the treatment on the animal’s health, which can be reflected in excessive weight gain or loss.^26^

##### 2.3.2.1 Histopathological Analysis of Mucosal Tissues

After the animal sacrifice, vaginal tissue was initially fixed in 4% (w/v) formaldehyde (Kaltek, Italy) and stored at room temperature. Then, vaginal tissue was embedded in paraffin wax, and sections of 20 μm were cut and placed on HistoBond+ coated microscope slides. After that, by processing in xylene, the tissues were deparaffinized and immersed in ethanol and water. Staining was carried out by the periodic-acid Schiff method (PAS). Then, the vaginal tissues were examined in a bright field using an Olympus BX51 epifluorescence microscope coupled to a DP72 digital camera (Olympus Portugal SA, Portugal) and the images were acquired using an Olympus Cell-B software.

*Candida*-free and infected vaginal tissues subsequently treated with OEO-KMPs were examined for histopathological changes in the mucosal tissues. The evaluation involved examining various factors, including epithelial lesions, glandular cystic dilatation, inflammatory infiltrates, hemorrhage, vascular proliferation/degeneration, edema/fibrosis, necrosis, and calcification in the vaginal epithelium. A scoring system was employed to quantify the severity of these factors, with scores ranging from 0 (no alterations) to 4 (very severe). These scores were then summed to determine the level of vaginal irritation, categorized as minimal (1–4), mild (5–8), moderate (9–11), or severe (12–16).^27^ The criteria for evaluation were based on standards outlined in the Biological evaluation of medical devices, particularly the tests for irritation and skin sensitization.^28^

### 2.4 Statistical Analysis

All results obtained during this study were statistically analyzed using the Prism software package (GraphPad Software version 8.0.1). In the microparticles characterization, one-way ANOVA and Tukes multiple comparison test were used to compare the different hours with the initial time. For the analysis of the OEO release rate, two-way ANOVA and Dunnett’s multiple comparisons tests were used to compare the different hours with the initial rate. The same test was used to compare the effect of the OEO-KMPs on biofilms versus the untreated biofilm. For all *in vitro* assays, three independent experiments were carried out and each analysis was performed in triplicate. In the *in vivo* assay, nonparametric test and Mann–Whitney test were used, and one of the points was removed as it was considered an outlier. The experiments were carried out with a confidence level of 95%. Statistical significance was assumed at *p* < 0.05.

## 3 Results and Discussion

### 3.1 Oregano Essential Oil Encapsulated into Microparticles of Keratin (OEO-KMPs)

The possible toxicity, strong taste, chemical instability, restricted administration routes and volatility of OEO limit its application.^29^ In fact, regarding EO toxicity, Janani et al.^30^ verified that cell viability with 50 μg mL^–1^ of OEO was 80%. However, from 100 and 200 μg mL^–1^, the presence of viable cells was less than 80%, demonstrating a cytotoxic effect from this concentration.^30^ To overcome these obstacles, nanoencapsulation technology is receiving particular attention. In fact, this technology allows EOs protection from light and temperature alterations, increases their solubility in aqueous environments, prolongs their release and improves their bioaccessibility and bioavailability.^31^ Electrospray, spray and precipitation methods have been used for the synthesis of micro and nanoparticles of keratin for drug delivery applications.^32^ In the present work, the OEO-KMPs were produced by ultrasound cycles through a high intensity ultrasonic and characterized regarding OEO-KMPs’ morphological and physicochemical parameters of the OEO-KMP, such as OEO encapsulation efficiency, particle stability (PDI, size, and surface charge), and the OEO release profile.

Encapsulation efficiency is a crucial characteristic for evaluating and validating nanocapsules as a delivery system.^33^ Indeed, a high OEO EE of 99.4 ± 0.1% was obtained, which clearly demonstrates the high capacity of wool keratin to load OEO during the homogenization process by ultrasound cycles. The EE of EOs using ultrasound homogenization methods varies widely between studies depending on the encapsulation material and techniques employed.^34^ In a similar process, Rajabinejad et al. prepared microcapsules using water-soluble keratin to encapsulate hydrophilic molecules and their yield was 83.6 ± 5%.^35^ On the other hand, Ma et al. encapsulated OEO in chitosan nanoparticles, using tripolyphosphate as a cross-linking agent, achieving an encapsulation efficiency of 92.9%.^36^ It should be noted that this method is a simple and versatile synthetic tool for micro or nanostructured materials.^37^ Moreover, microcapsules formed from keratin have shown attractive properties, such as high excellent biocompatibility, surface area, biodegradability and easy functionalization, being a promising option for controlled and targeted administration of compounds.^38^ Furthermore, the significant hydrogen and disulfide bonds confer to keratin some mechanical properties, such as rigidity, stability, strength and resistance to proteolytic degradation.^17^

**Figure 1:**
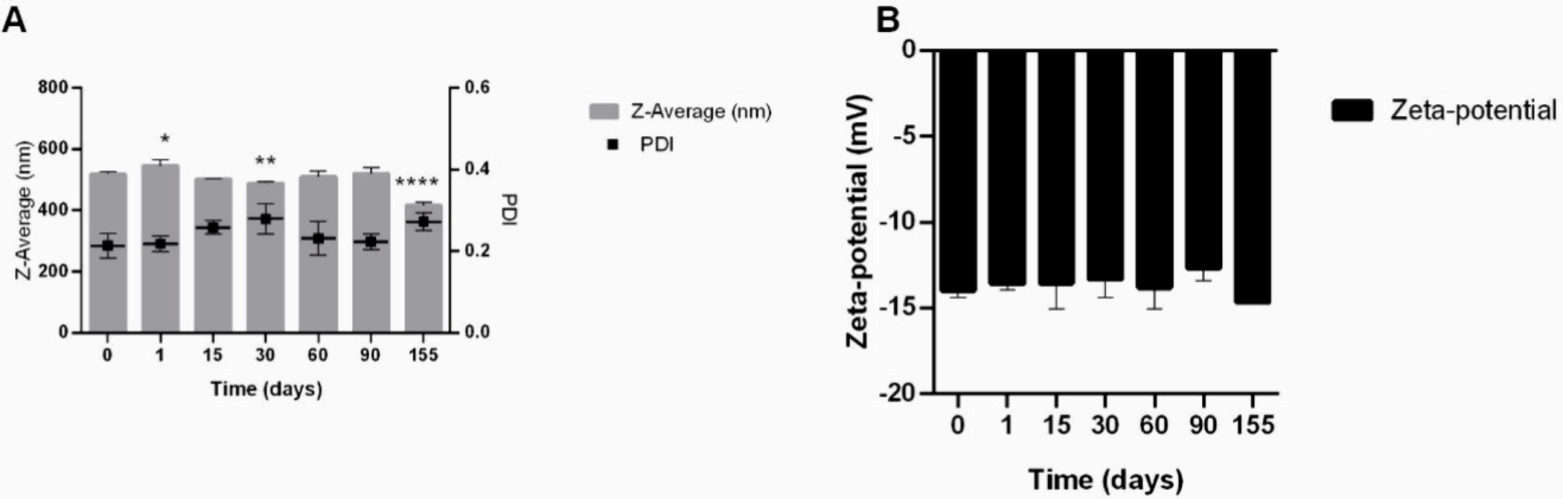
Characterization of keratin nanoparticles prepared in PBS encapsulating oregano oil (OEO-KMPs) for 5 months (155 days). (A) particle size (Z-average) and polydispersity (PDI); (B) surface charge (zeta-potential). * indicates statistical difference in particle size when compared to the results obtained at time 0 (initial conditions) (*p < 0.1, **p < 0.01, ****p < 0.0001).

The proper functioning of the micro or nano delivery systems is also greatly influenced by their size, shape, dispersion and surface chemistry.^39,40^ In addition, the ability of microcapsules to retain the OEO can vary depending on time and storage conditions. Due to the importance of these factors in OEO delivery application, this study evaluated the OEO-KMPs’ properties over time (Figure 1).

Although there was a slight statistical difference (*p* < 0.01) on days 1 and 30, and a significant difference (*p* < 0.0001) between day 1 and day 155, the OEO-KMPs’ particles exhibit a mean size (Z-average) of approximately 500 nm over 90 days of storage. These differences observed might be related with some rearrangements on the protein network on OEO-KMPs surface.^41^ The small dimensions of particles enable them to cross diverse biological barriers, facilitating the transport of drugs to various levels within the microorganism.^40^ In fact, as particle size decreases, specific surface area, reactivity, and the bioavailability of encapsulated drugs increase, leading to an amplified functional capacity of the bioactive agent, remarkably improving antimicrobial efficacy.^40^

The PDI serves as a metric to evaluate the uniformity of the particle sizes. Relatively to the values of the OEO-KMPs, the values of this parameter were between 0.2 and 0.3, without a significant difference for 155 days. A PDI value below 0.3 indicates a well-proportioned size distribution in a colloidal system, minimizing the risk of precipitation.^40,42^

Zeta potential is another crucial parameter. In addition to being a good indicator of the stability of nanoparticles in suspension, due to the extent of electrostatic repulsion/attraction between particles but also as an indicator of particle involvement with biological systems.^40^ Relatively to OEO-KMPs, this parameter also remained stable over time, between −14 and −15 mV due to the carboxyl groups within keratin.^12^ From the standpoint of colloidal stability, a colloidal suspension highly negative is considered stable, the repulsive forces prevent agglomeration, thus contributing to the overall stability of the system.^14^ Therefore, based on these three parameters, it can be concluded that the OEO-KMPs prepared are stable and uniform in neutral aqueous media (PBS).

**Figure 2:**
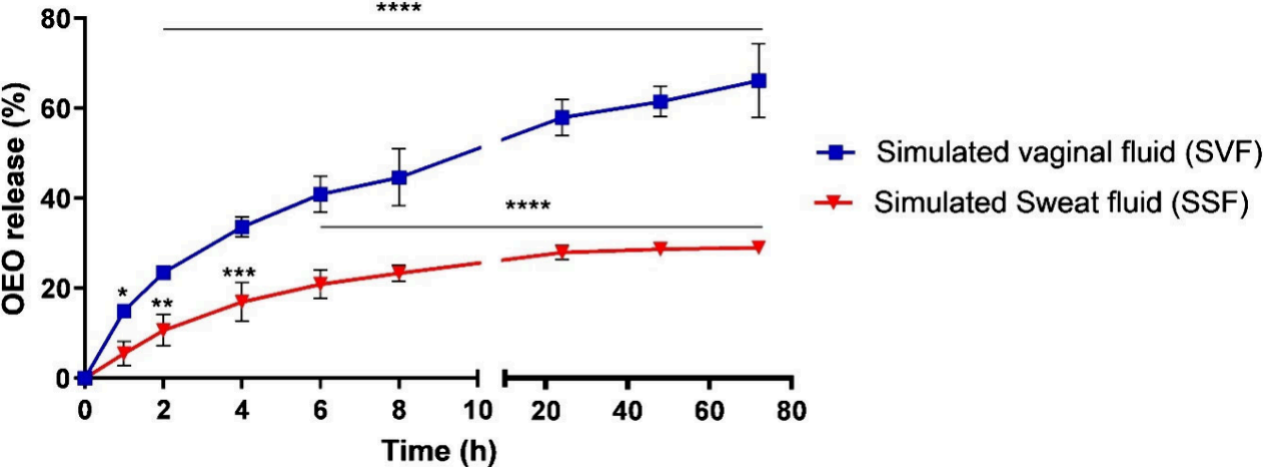
*In vitro* release profiles of oregano essential oil from keratin-based particles in simulated vaginal and sweat fluids, over 72 h. * indicates statistical difference compared to the results obtained at time 0 h (*p < 0.1, **p < 0.01, ***p < 0.001, ****p < 0.0001).

The selection of the EO, along with the decision regarding the delivery system, impacts the achieved therapeutic.^40^ A good drug delivery system should be able to encapsulate molecules and release them over time under physiological conditions.^15^ The *in vitro* release profiles of OEO encapsulated into the keratin-based particles was studied by incubating the OEO-KMPs in SVF and SSF for 72 h (Figure 2).

The OEO release reached approximately 50% and 20% in the first 8 h of incubation in SVF and SSF, respectively (Figure 2). After 72 h of incubation, there was a maximum OEO release of 65% in SVF and 30% in SSF. These findings are in accordance with previous studies by Ma et al.^36^ and Sotelo-Boyas et al.,^43^ which demonstrated a biphasic release profile characterized by an initial intermittent release followed by slow release over an extended period, allowing for gradual release of the oil over several days. In Ma et al.’s study, there was an initial rapid release of 41.27 ± 0.56% within the first 5 h, followed by continuous release totaling 82.73 ± 1.53% over the subsequent 8 days.^36^

The difference obtained between SVF and SSF can be related to the affinity of the OEO compounds with the release medium, allowing a higher and faster rate of oil release.^43,44^ The release rate of active compounds from polymeric nano/microcapsules is dependent on several factors which may be related to the properties of the substance incorporated in the microcapsules, such as solubility or desorption of the surface-bound or adsorbed substance, or the matrix properties of the nanocapsules such as the diffusion of the substance through the matrix, erosion or degradation/disintegration of the matrix, penetration of the release medium through the matrix or the combination of erosion and diffusion processes.^43,45^ Moreover, it was observed that the OEO release profiles showed an increased effect in the first hours of incubation before reaching a stable release profile. The higher initial release can be attributed to the OEOs’ molecules adsorbed on the particle surface and to the EO entrapped near the surface of the matrix, since the rate of dissolution of the substance near the surface is high, the amount of substance released will be also high in the first few hours.^36,43,45,46^

In addition, the stability of the OEO-KMPs was evaluated after 72 h of contact with both fluids (SSF and SVF) and it was observed that the size of the OEO-KMPs showed significant differences when in contact with the SVF (*p* < 0.0001) (Table 1) compared with OEO-KMPs in PBS at time 0 (Figure 1). This may indicate that the particles when in contact with SVF undergo structural changes, allowing the release of OEO. Moreover, the differences observed on particle sizes when incubated in both fluids can be related to the fluids’ pH, since SVF has the lowest pH (4.2) compared to SSF of 5.5. According to Yang et al.,^47^ adjusting the pH of a solution allows keratin to function as a polycation or as a polyanion. Specifically, when the pH of the solution is lower than the isoelectric point of keratin, the net charge of the keratin molecule reverts to positive.^47^ This phenomenon is common among proteins and biopolymers, where the charge is influenced by the ionization states of acidic and basic groups within their structure. pH adjustments can induce protonation or deprotonation of these groups, thus changing the overall charge of the molecule.^47,48^

**Table 1:** Characterization of Keratin Nanoparticles Prepared in PBS Encapsulating Oregano Oil (OEO-KMPs) when in Contact with Simulated Vaginal Fluid and Simulated Sweat Fluid for 72 h^a^

	Particle size (Z-average nm)	PDI	Zeta- potential (mV)
Phosphate-buffered saline	517.8 ± 8.09	0,213 ± 0.03	–14.00 ± 0.40
Simulated vaginal fluid	1809.00 ± 273.41****	0.347 ± 0.20	–12.55 ± 0.14
Simulated sweat fluid	484.55 ± 14.83	0.336 ± 0.02	–14.05 ± 0.50

a Asterisk (*) indicates statistical difference in particle size when compared to the results obtained at time 0 (Figure 1) (****p < 0.0001).

**Figure 3:**
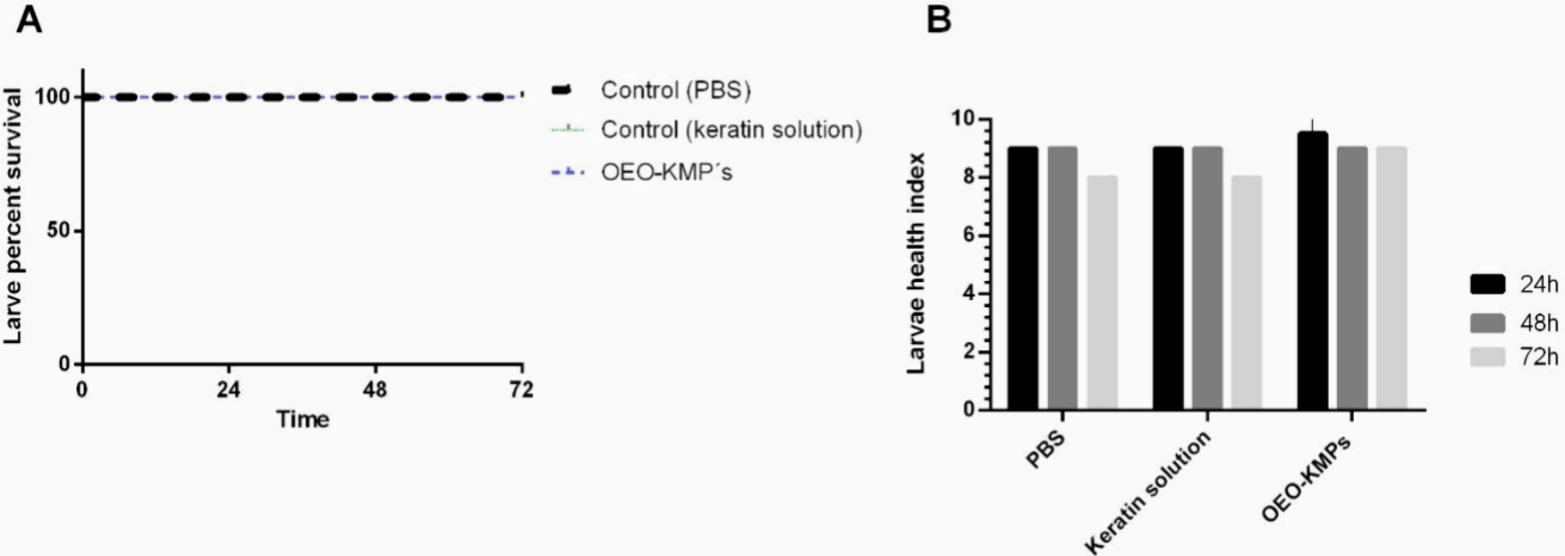
Keratin microparticles encapsulating oregano oil (OEO-KMPs) cytotoxicity measured in *Galleria mellonella* model. (A) Survival curves of *G. mellonella* larvae in contact with OEO-KMPs and respective controls (without exposure to OEO-KMPs and keratin solution) during 72 h; and (B) *G. mellonella* larvae health index after 24, 48, and 72 h in contact with OEO-KMPs and respective controls.

Having analyzed the stability of the OEO-KMPs, it becomes essential to evaluate the cytotoxicity of this formulation. To this end, the response resulting from the interaction between the OEO-KMPs and the surface of the *G. mellonella* larva body was examined. Similar to the prior investigation involving VP-OEO,^6^ there was no evidence of toxicity after 72 h of larval contact with either keratin solution or OEO-KMPs’ formulations in this current research. Indeed, all larvae maintained their viability up to 72 h (Figure 3.A), and there were no significant differences in the larval health index (Figure 3.B) following contact with PBS, keratin solution, or OEO-KMPs. Hence, it can be concluded that the application of OEO-KMPs is considered safe without manifesting any surface irritation signal. Several studies have indicated a reduced occurrence of side effects with nanostructured systems, which incorporate lower drug doses without compromising therapeutic efficacy.^49−51^ Consequently, these nanocarriers have been explored for diverse applications to address the limitations associated with conventional formulations.^49^

### 3.2 Impact of OEO-KMPs on *C. albicans* Growth *in Vitro*

The high incidence of *C. albicans*, the therapeutic limitations, and the associated negative consequences make it crucial to develop effective alternatives to the current treatments. In this context, the previous work demonstrates that the application of VP-OEO is safe and effective against both *C. albicans* and *C. glabrata*, inducing changes in membrane integrity and metabolic activity.^6^ In this work, we tested the encapsulation of this EO into keratin microparticles, allowing a more controlled and safer release of the OEO.

As previously mentioned, the OEO-KMPs have dimensions of approximately 500 nm and several studies corroborate that particles with dimensions around 500 nm facilitate the penetration of delivery systems into fungal cells.^52^ The inhibitory activity of the OEO-KMPs on the planktonic growth of the *C. albicans* was evaluated using the disk-diffusion agar method, and the results showed a zone of inhibition of 17.3 ± 0.28 mm. The keratin solution evidently had a weak impact on the growth of fungal cells when cultured in solid medium. Despite this, employing the broth microdilution method, it was observed that the use of OEO-KMPs resulted in complete inhibition (6–7 Log reduction) of *C. albicans* in its planktonic state. These findings indicate that OEO-KMPs exert a more pronounced effect when cells grow in liquid medium. The release rate of OEO-KMPs may vary depending on the conditions of the culture medium, probably requiring a liquid medium to destabilize the structure of the particles and release the compounds that interact with the microorganisms, which does not occur when placed in a solid medium.

**Figure 4:**
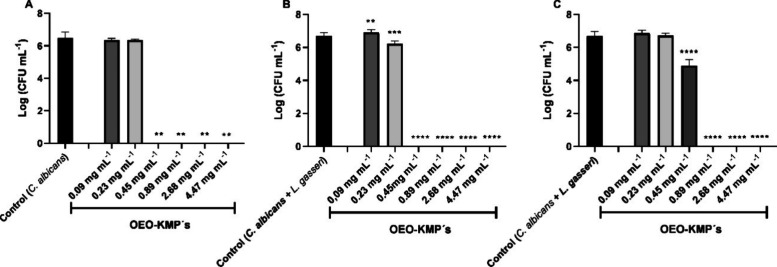
Effect of the keratin microparticles encapsulating oregano oil (OEO-KMPs) on single and mixed infection of *Candida albicans* with *Lactobacillus gasseri*. (A) Single infection of *C. albicans*; (B) Mixed infection (*C. albicans*); (C) Mixed infection (*L. gasseri*)*.* * indicates statistical reduction of biofilms cell cultivability in comparison with the respective control (**p < 0.01, *** *p* < 0.001, **** *p* < 0.0001).

*Candida* infections are often associated with biofilm formation, leading to resistance development and, consequently, the need for more potent therapies that can induce changes in beneficial vaginal microbiota. It is therefore essential to confirm that this alternative treatment does not cause an imbalance in the remaining microbiota. Indeed, beneficial bacteria play an important protective role in prevention gynecological diseases. In our previous work, we observed that the vapor phase of white thyme EO (VP-WTEO) had a significant inhibitory effect on the number of resistant *C. albicans* cells colonizing a reconstituted human vaginal epithelium (RHVE) without changing the number of *L. gasseri* cells, making this application a safe alternative for the remaining vaginal microbiota. Despite the indication on the safety of VP-WTEO toward *L. gasseri*, it was important to evaluate if OEO, being more potent, might exert a more pronounced impact on *Lactobacillus* species. The OEO-KMPs’ concentration that inhibits/damages *C. albicans* biofilms but does not influence the permanence and viability of *Lactobacillus* species was determined. To this end, the effect of the OEO-KMP in the OEO concentration range 0.09–4.47 mg mL^–1^ on single biofilms of *C. albicans* (Figure 4.A) and on mixed biofilms of *C. albicans* (Figure 4.B) and *L. gasseri* (Figure 4.C) was evaluated. In both types of biofilms, single or mixed, the effect of OEO-KMPs followed the same pattern, with the concentration of 0.45 mg mL^–1^ eradicating completely (*p* < 0.01) the *C. albicans* biofilm. So, the results demonstrated that only 2.5% of OEO-KMPs corresponding to 0.45 mg mL^–1^ of OEO encapsulated allows to eradicate *C. albicans* mature biofilms and preserve the *L. gasseri*, at the same time (Figure 4). Therefore, a safe concentration for eradicating *C. albicans* biofilms has been determined at 0.45 mg mL^–1^ of the OEO-KMPs. Fluconazole, used as a reference point, revealed an MIC of 0.25 μg mL^–1^ when exposed to *C. albicans* SC5314 biofilm cells at pH 4, similar to vaginal fluid.^53^ However, recent studies have revealed greater resistance of this species to fluconazole.^54^ This suggests that OEO-KMPs may have advantages, particularly considering fluconazole’s potential decreased effectiveness due to increased resistance.

### 3.3 *In Vivo* Effect of OEO-KMPs on *C. albicans* Infection

**Figure 5:**
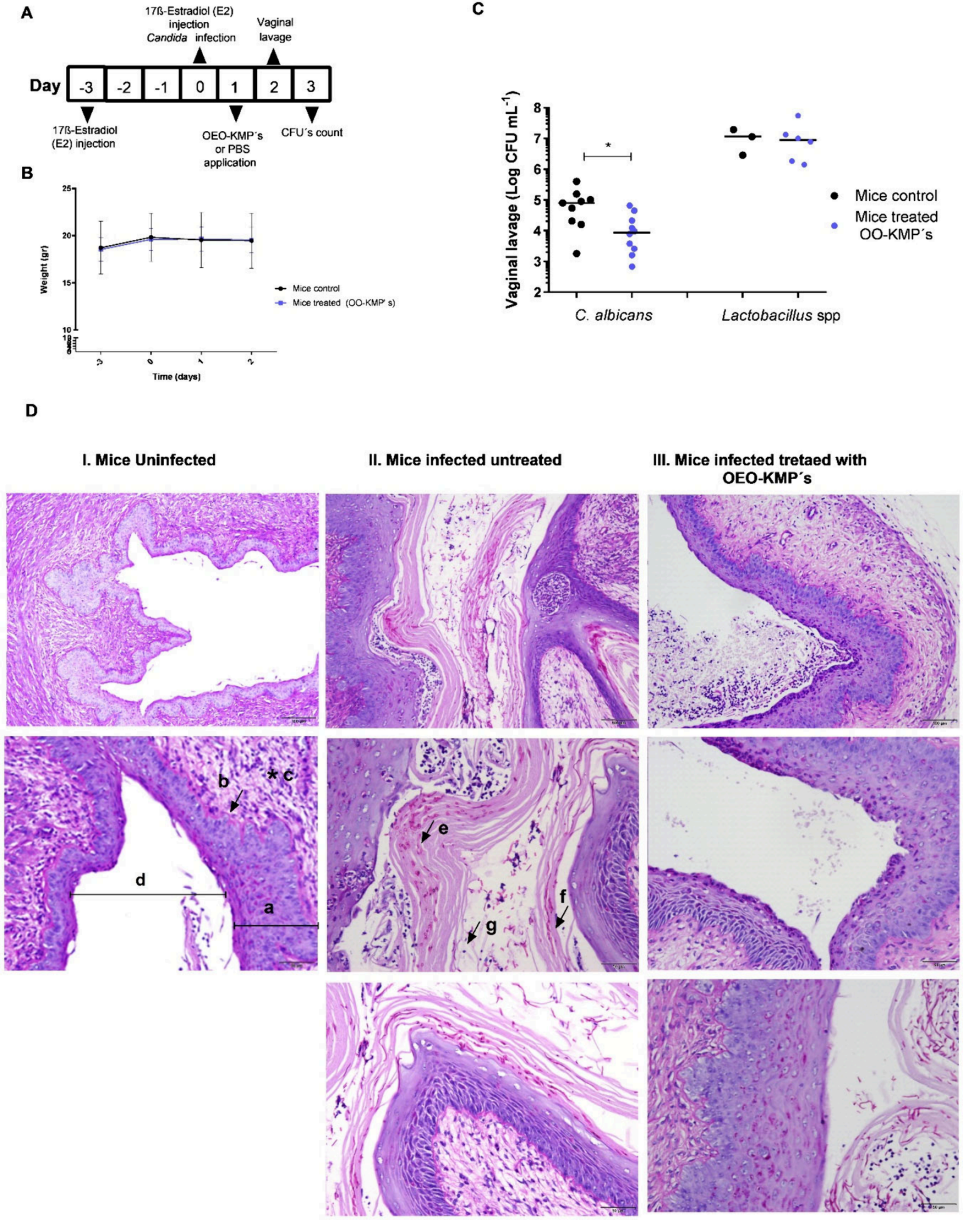
Effect of OEO-KMPs on mice model of vaginal candidiasis. (A) Timeline of *Candida albicans* infection and OEO-KMPs’ treatment of mice; (B) Mice weight according to the timeline of infection; (C) *C. albicans* and *Lactobacillus* cells detected in mice vaginal lavage fluid (Log CFU mL^–1^) represented is the median with 95% confidence interval (*p < 0.05); (D) Histological images of vaginal tissue stained with periodic acid-Schiff (PAS), increasing magnification along the column: I. Mice uninfected (negative control); II. Mice infected with *C. albicans*, untreated (positive control); III. Mice infected with *C. albicans*, treated with OEO-KMPs for 24 h after infection. (a) Layer of superficial squamous epithelial cells, (b) basal membrane, (c) lamina propria (d) vaginal lumen, (e) and (f) *C. albicans* cells and (g) neutrophils. The results were registered under 10× and 20× magnification. The data presented in this study are representative of at least two independent experiments.

A well-designed vaginal drug delivery system, based on the use of microparticles encapsulating the medication, should ensure uniform distribution throughout the vaginal cavity, prolonged retention at the administration site, and sustained release of the drug, while avoiding adverse effects on the local vaginal epithelium.^49,55^ In this sense, the mouse vaginal model is an appropriate tool to mimic human vaginal conditions as much as possible and to evaluate new therapies, as well as to verify the host’s defenses against fungal infections.^56,57^ The efficacy of OEO-KMPs *in vivo* was evaluated using an experimental VVC mouse model (Figure 5.A). For this, a mouse model with VVC was established with the support of 17β-Estradiol, a hormone which is an effective precipitator of VVC.^56,58,59^ Female BALB/c mice were infected intravaginally with *C. albicans,* and the dose of estradiol was reinforced, to maintain the ideal conditions for inducing infection (Figure 5.A). After 24 h of infection, the OEO-KMPs or PBS (control group) were applied intravaginally, and the formulation were allowed to contact with the localized infection during 24 h, as shown in Figure 5.A. After this time, vaginal lavages were performed and the amount of *C. albicans* and *Lactobacillus* species in the vaginal canal were quantified by CFU counting. The mice were then sacrificed, and the vaginal tissues isolated and analyzed under a microscopy. The body weight of each animal, an important parameter that allows the detection of side effects of the treatment on the animal’s health, which can be reflected in excessive weight gain or loss, was monitored over time.^26^ Throughout the entire procedure, infection, and subsequent treatment, there were no significant changes in the body weight of the treated animals compared to the control group, with the same pattern being observed over time (Figure 5.B).

The results of the CFUs in vaginal lavages showed a significant difference (*p* < 0.1) between the treated group of animals and the untreated animals after 24 h of OEO-KMPs’ application. In fact, a single intravaginal application of OEO-KMPs (0.69 mg of OEO) induced a 1 Log CFU mL^–1^ reduction in *C. albicans* cells compared with control (untreated mice). This result suggests that a single intravaginal application of the OEO-KMPs is able to control the growth of *C. albicans* in an environmental niche.

A downward trend in *Candida* infection is evident, despite the dispersion in *in vivo* assays that can be considered usual, as it entails several factors that can influence the initial infection and subsequent treatment, mainly the hormonal cycle of female mice. During the menstrual cycle, the female reproductive tract is highly sensitive to changing sex hormones.^59,60^ In women, the menstrual cycle lasts approximately 28 days and consists of three main phases: (a) follicular phase, (b) ovulatory phase, and (c) luteal phase; in rodents this cycle, called the estrous cycle, lasts approximately 4–5 days and the 3 corresponding phases are called (a) proestrus, (b) estrus and (c) metestrus.^59,60^ During the different phases of the cycle, the vaginal epithelium changes, and in the luteal phase it keratinizes and hardens, resulting in epithelial thickening and desquamation during menstruation.^58^ Indeed, hormonal effects may have implications for the development and treatment of VVC, the recurrence of VVC is frequently observed during pregnancy and in the late luteal phase, occurring just prior to menstruation.^61^ Kalo-Klein et al.^62^ observations revealed that *C. albicans* filamentation and consequently infection was maximal during the luteal phase. Gonçalves et al.^61^ also reported that *C. albicans* is susceptible to hormone-induced biofilm dispersion. Therefore, controlling the estrous cycle in mice can be challenging due to their relatively rapid cycle, even with the administration of 17β-Estradiol.

Moreover, one of the crucial aspects of this therapy is that it preserves the remaining microflora, in particular, the *Lactobacillus* species, as shown in Figure 5.C. A single intravaginal application of the OEO-KMPs does not seem to disrupt the population of the *Lactobacillus* population present in the murine microflora, which remains at a concentration of approximately 1 × 10^7^ CFU mL^–1^ without significant differences compared to untreated mice. As mentioned above, this factor is imperative and it is worth noting that bacteria are the main component of the human microbiome and beneficial bacteria play a fundamental role in maintaining fungi in a commensal state, acting as the first line of defense against fungal infection.^63^

To better understand the effect induced by the treatment with the OEO-KMPs in the vaginal environment, we histologically examined the vaginal tissues were histologically examined. Thus, vaginal tissues of healthy animals (negative control) were compared with infected untreated (positive control) and treated animals (Figure 5.D). The vagina is a fibromuscular canal; in its relaxed state, the vaginal wall collapses and obliterates the lumen and the epithelium appears “pleated”. This fibromuscular canal contains the following layers: mucosal layer: squamous stratified epithelium (mucosal layer) (Figure 5.D (a)); lamina propria: dense connective tissue, rich in elastic fibers, well vascularized (Figure 5.D (c)); muscular layer: smooth muscle and adventitial or serous layer: lax connective tissue. The oval structures and red filaments seen in the vaginal tissue images were identified as *C. albicans* cells (Figure 5.D (e and f)).

Potential tissue damage from irritation includes hyperplasia, necrosis, changes in the stratum lucidum and corneum of the vaginal epithelium, detachment and separation of cells, vascular changes or damage to the vessel walls (arterioles and capillaries).^64−66^ The images revealed that the vaginal tissues infected with *C. albicans* (positive control) possessed a notable production of keratin by squamous cells, with identifiable mycotic forms, coupled with an intense exocytosis of the epithelium by neutrophils, sometimes (or frequently) aggregated on the surface of the epithelium with the formation of subcorneal pustules. Moreover, it is possible to observe the vaginal lumen with intense desquamation (desquamated cells and keratin remains) and with accumulation of neutrophils (Figure 5.D). Furthermore, the images show a decrease on vaginal tissue lesions with the treatment of mice with OEO-KMPs, resulting in a decrease of the accumulation of inflammatory cells (neutrophils) and in formation of a superficial layer thinner with a clear reduction in intense desquamation (sloughed cells and keratin debris) in the vaginal lumen (Figure 5.D).

According to the literature, these layers are composed of bundles of keratin intermediate filaments, which form as epithelial cells differentiate and die. The most superficial keratinized layers are only loosely attached and are constantly lost (shedding) and replaced. As cells in the deeper (basal) layer divide and move toward the surface, they undergo several morphological and biochemical changes and when they reach the surface, they undergo a process of keratinization. This process is important as it helps to support the surface layer of the vaginal epithelium, making it more resistant to mechanical damage, variations in pH, and infectious agents.^64^ In this study, after *Candida* infection, this mechanism is observed, so it could be a protective mechanism together with neutrophils. Despite the injection of estradiol, neutrophils appear to be present in the vaginal lumen (Figure 5.D). Moreover, the presence of cells of microorganisms smaller than the *Candida* species is evident. These microorganisms probably correspond to bacteria naturally present in the vaginal fluid of mice, potentially including the lactobacilli, consistent with the results obtained concerning *Lactobacillus* persistence (Figure 5.C). While variations in tissue damage and the presence of *Candida* cells were observed within the same group (a phenomenon considered common in *in vivo* assays), it is notable that fewer *Candida* cells were associated with an increase of small structures, potentially indicative of *Lactobacillus*, as discussed earlier.

To support these conclusions, the assessment of vaginal irritation after the experimental procedure in the different groups was based on a histopathological evaluation of four parameters in the vaginal tissue: epithelial lesions, inflammatory infiltrates, vascular congestion, and edema/fibrosis (Table 2).^27^

**Table 2:** Histopathological Evaluation of Vaginal Irritation after Infection and 24 h of Rectovaginal Application of PBS (Positive Group) or OEO-KMPs^a^

Parameters	Healthy tissue (negative control)	Infected tissue (positive control)	Infected tissue and treated with OEO-KMPs
Epithelial lesions	0	3	1
Inflammatory infiltrates	0	3	3
Vascular congestion	0	2	0
Edema/fibrosis	0	1	0
Total score	0	9	4

a Score interpretation: minimal (1–4), mild (5–8), moderate (9–11), and severe (12–16).

Healthy tissue exhibits stratified squamous epithelium with dense subepithelial connective tissue with no sign of damage, obtaining a score of 0. In the untreated infected group, a score of 9 (moderate) was obtained, primarily due to inflammatory infiltration and remnants of necrotic tissue. The tissue from mice treated with the OEO-KMPs obtained a score of 4 indicating minimal damage, which is considered suitable for clinical trials of vaginal products.^27^ However, there is an accumulation of inflammatory cells associated with *C. albicans* infection. Inflammation of the reproductive tissue is assessed by the magnitude of inflammatory infiltrates such as neutrophils, macrophages, and lymphocytes.^67^ These results suggest that while a single application of OEO-KMPs can be used safely, the presence of inflammatory cells highlights the ongoing need for repeated treatment to address the inflammation associated with *C. albicans* infection. In fact, the potential expulsion of therapy, influenced by the dynamics of vaginal fluid and the self-cleaning action of the vaginal tract, can lead to incomplete administration of sufficient doses.^49,55^ Consequently, multiple doses/day are often necessary to achieve the desired therapeutic effects.^49,55^

Furthermore, it is crucial to recognize that the mouse model of vaginal candidiasis, while a valuable research tool for studying vaginal candidiasis due to its similarities to the chronic nature of the disease in women, still exhibits differences from human vaginal candidiasis in several physical aspects. These distinctions include the lack of *Candida* species as part of the vaginal microbiota, requiring exogenous estrogen to initiate fungal colonization, and differences in vaginal pH.^24,68^ Consequently, it is essential to evaluate this therapy in alternative animal models, potentially including larger species, to obtain a more comprehensive understanding of their potential effects in cases of VVC in women.

## 4 Conclusions

This study confirms the *in vitro* and *in vivo* efficacy of OEO-KMPs against *C. albicans* infection. Furthermore, a key aspect of the formulations is their stability, which has been confirmed over time. These findings constitute an important step toward understanding the potential of OEO-KMPs as antifungal agents. Our research demonstrated that a single application of OEO-KMPs effectively reduced *C. albicans* levels in an *in vivo* vaginal model while maintaining the *Lactobacillus* spp. population, providing the theoretical basis for the potential future clinical use of OEO-KMPs as antifungal agents, which may have a less harmful effect on women’s health. The choice of this delivery method (KMP) aligns with the natural origin of the OEO and its potential to minimize adverse effects. Therefore, one possible application of this therapy is its incorporation into a topical lotion for vagina use. It is worth noting that although the *in vivo* study in a murine model was successful, further investigation should address the diversity of *Candida* strains and consider the possibility of mixed infections and clinical settings in order to obtain a more complete understanding of the therapeutic potential of OEO-KMPs.

This research emphasizes the potential of OEO-KMPs as a novel, natural, and effective antifungal treatment, opening the door to safer and more effective approaches to treating *C. albicans* infections in women’s health.

### Ethics Approval and Consent to Participate

All animal procedures conformed to EU Directive 2010/63 EU and national regulations (RD 53/2013). All animal procedures were approved by the HGUGM. Animal Experimentation Ethics Committee, the local Ethics Committees, and the Animal Protection Board of the Comunidad Autónoma de Madrid (PROEX 083/18).

## Data Availability

Not applicable.

## Abbreviations

ATCC: American Type Culture Collection
CFU: colony forming units
CLSI: Clinical and Laboratory Standards Institute
EE: encapsulation efficiency
EOs: essential oils
KMPs: keratin microparticles
OEO: oregano essential oil
OEO-KMPs: oregano EO-loaded keratin microparticles
PBS: phosphate buffer saline
PDI: polydispersity index
SDA: Sabouraud dextrose agar
SDB: Sabouraud dextrose broth
SDS: sodium dodecyl sulfate
SSF: simulated sweat fluid
SVF: simulated vaginal fluid
VP-EOs: vapor phase of essential oils
VP-OEO: vapor phase of oregano essential oil
VP-WTEO: vapor phase of white thyme essential oil
VVC: vulvovaginal candidiasis
E2: 17β-Estradiol.
